# Lower limb muscle strength is associated with functional performance and quality of life in patients with systemic sclerosis

**DOI:** 10.1590/bjpt-rbf.2014.0084

**Published:** 2015-03-13

**Authors:** Tatiana R. L. Lima, Fernando S. Guimarães, Mara N. Carvalho, Thaís L. M. Sousa, Sara L. S. Menezes, Agnaldo J. Lopes

**Affiliations:** 1 Programa de Pós-graduação em Ciências da Reabilitação, Centro Universitário Augusto Motta (UNISUAM), Rio de Janeiro, RJ, Brazil; 2 Departamento de Fisioterapia, Universidade Federal do Rio de Janeiro (UFRJ), Rio de Janeiro, RJ, Brazil; 3 Serviço de Pneumologia, Hospital Federal de Bonsucesso, Rio de Janeiro, RJ, Brazil; 4 Programa de Pós-graduação em Ciências Médicas, Universidade do Estado do Rio de Janeiro (UERJ), Rio de Janeiro, RJ, Brazil

**Keywords:** systemic sclerosis, muscle strength, nutritional status, respiratory function tests, exercise, rehabilitation

## Abstract

**Background:**

Complaints of peripheral muscle weakness are quite common in patients with systemic sclerosis (SSc). It is likely that the muscle impairments may reduce the patients' exercise performance, which in turn may decrease their functional capacity and exert a direct impact on their quality of life.

**Objectives:**

To assess the peripheral and respiratory muscle strength in individuals with SSc and to investigate their correlation with the 6-min walk distance (6MWD) and quality of life measurements. Moreover, we aimed to characterize their nutritional status, pulmonary function, functional capacity, and quality of life compared to the controls.

**Method:**

The present cross-sectional study included 20 patients with SSc and 20 control subjects. All of the participants were subjected to isometric dynamometry, surface electromyography, bioelectrical impedance analysis, pulmonary function testing, and the 6-min walk test. Patients with SSc also responded to the Medical Outcomes Study 36-Item Short-Form Health Survey (SF-36) and the Health Assessment Questionnaire Disability Index (HAQ-DI).

**Results:**

The individuals with SSc exhibited a reduction in quadriceps strength (p=0.0001), increased quadriceps fatigability (p=0.034), impaired pulmonary function, and a reduced 6MWD (p=0.0001) compared to the controls. Quadriceps strength was significantly correlated with the 6MWD (*Rho*=0.719; p=0.0004) and the HAQ-DI (*Rho*=-0.622; p=0.003). We also found significant correlations between quadriceps fatigability and maximal inspiratory (*Rho*=0.684; p=0.0009) and maximal expiratory (*Rho*=0.472; p=0.035) pressure.

**Conclusions:**

Patients with SSc exhibited reduced respiratory muscle and quadriceps strength and an increase in its fatigability. In these individuals, there was a relationship between quadriceps strength, functional capacity, and quality of life.

## Introduction

Systemic sclerosis (SSc) is a connective tissue disease characterized by skin and organ fibrosis, vascular obliteration, immune cell activation, and autoimmunity^1^^,^ 2. Its incidence is close to 20 cases per million per year, and its prevalence is approximately 10 times higher^1^. SSc occurs two to eight times more often among females compared to males^2^. Involvement of the upper limbs is predominant, although SSc is a multisystemic condition associated with functional impairments^3^. In addition to the skin affection, which can be quite disseminated and disabling, SSc can also affect multiple organs and systems, including the lungs, musculoskeletal system, kidneys, heart, and gastrointestinal tract, which worsens prognosis^1^^-^ 4.

Due to the multisystemic nature of SSc, several factors may potentially limit the functional capacity of individuals affected with SS, including cardiopulmonary compromise, osteoarticular abnormalities, and musculoskeletal disease^5^^,^ 6. It is likely that these impairments may reduce the patients' exercise performance, which in turn may decrease functional capacity and cause a negative impact on the quality of life^4^^,^ 5.

Muscle affection in SSc has been minimally investigated^4^. Its reported prevalence varies from 7% to 81%, thus reflecting the wide heterogeneity of the criteria by which it is defined^7^^-^ 9. The muscle alterations most frequently reported in SSc patients are inflammatory and non-inflammatory myopathy, which most often occur in areas close to the affected joints^9^. In some patients with SSc, the joint contractures and the chronic nature of the disease result in disuse weakness and atrophy of the skeletal muscles. In addition, drug-induced muscle disease must be taken into consideration whenever muscle weakness appears concomitantly with the use of therapeutic agents known to cause myopathy, such as D-penicillamine, glucocorticoids, and antimalarials^8^^,^ 9.

Muscle weakness is a common occurrence and is reported by more than 80% of patients with SSc^7^^,^ 8. Using a four-point scale, Clements et al.^8^ detected muscle weakness in 30% of cases. In turn, Medsger Jr et al.^7^ found a reduction of >75% in muscle strength relative to normal performance in 43% of patients. More objectively, Azevedo et al.^10^ found that in 100% of patients, the mean peak torque of the elbow muscles was lower than expected as measured by isokinetic dynamometry. The 6-min walk test (6MWT) is a simple test to assess submaximal exercise capacity that is safe, inexpensive, noninvasive, highly reproducible, and reflects the effort required to perform daily living activities^11^. Despite its feasibility, the 6MWT is non-specific, making it difficult to identify which factors are responsible for the functional impairment. In this way, the contributions of physical deconditioning, nutritional status, joint impairments, cardiopulmonary disease and peripheral muscle dysfunction for the functional disabilities in SSc have not been well documented^10^^,^ 12. Thus, we hypothesize that lower limb muscle function may significantly influence performance on the 6MWT in individuals with SSc. The objectives of the present study were to assess peripheral and respiratory muscle strength and endurance in individuals with SSc and to investigate their correlation with the 6MWT outcome and quality of life measurements. Moreover, we aimed to characterize their nutritional status, pulmonary function, functional capacity, and quality of life in comparison with a sample of healthy subjects. Given the scarcity of studies on the functional aspects of SSc, we believe that these results may subsidise future interventional studies on the rehabilitative strategies for these patients.

## Method

### Patients

This cross-sectional study was conducted between April 2013 and January 2014 and included 31 consecutive patients with SSc recruited at Hospital Federal de Bonsucesso and Hospital Federal da Lagoa, Rio de Janeiro, RJ, Brazil. Adults (18 years old or older) were diagnosed with SSc based on the criteria formulated by the American College of Rheumatology/European League Against Rheumatism^13^. The patients were categorized as having either limited or diffuse SSc^13^. The onset of disease was defined as the date when the first symptom of SSc appeared, which is usually Raynaud's phenomenon^13^. The exclusion criteria were peripheral oxygen saturation (SpO2) <90% at rest, a history of orthopaedic surgery of the chest or lower limbs, and difficulty in walking.

A control group of 20 healthy volunteers (19 females) was recruited from Centro Universitário Augusto Motta (UNISUAM), Rio de Janeiro, RJ, Brazil. These individuals did not have a history of smoking and did not exhibit any evidence of cardiorespiratory or musculoskeletal disorders.

All of the participants signed an informed consent form, and the protocol was approved by the Research Ethics Committee of UNISUAM, under protocol number 300.826/2013.

### Assessment of quality of life

The questionnaire known as the Medical Outcomes Study 36-Item Short-Form Health Survey (SF-36) was used to assess quality of life. This questionnaire comprises eight domains (physical functioning, physical role, bodily pain, general health perception, vitality, social functioning, emotional role, and mental health) that may be summarized into a physical and a mental component measure. The SF-36 total score may vary from 0 (poorest health status) to 100 (best health status). This questionnaire was translated into and validated in Portuguese^14^.

### Assessment of body composition

Body composition was assessed using a bioelectrical impedance analysis device (BIA 310e, Biodynamics, Seattle, WA, USA). Individuals were instructed to rest for 5 min before the test. They then stood barefooted with their feet 15 to 30 cm apart, away from any metallic objects^15^. Two electrodes were placed on the back of the right hand, and two others were placed on the top of the right foot. Resistance and reactance were calculated and then used to estimate the FFM using an equation previously validated for Brazilians: FFM = -4.104 + (0.518 × height^(2)^/resistance) + (0.231 × weight) + (0.130 × reactance) + (4.229 × gender: male = 1, female = 0)^15^. Two estimates of fat-free mass (FFM) were made for each subject, and the results were averaged^15^.

### Assessment of pulmonary function

Pulmonary function testing consisted of spirometry, carbon monoxide diffusing capacity (DLco), and respiratory muscle strength. Measurements were conducted using an HDpft 3000 (nSpire Health, Inc., Longmont, CO, USA) following standard procedures and interpretation^16^. After three acceptable forced vital capacity (FVC) maneuvers have been obtained, the maneuver with the largest sum of FVC and forced expiratory volume during the first second (FEV_1_) was selected for interpretation. For DLco measurement, the average of two acceptable tests was reported. For maximal inspiratory pressure (MIP) and maximal expiratory pressure (MEP), the largest value from three acceptable efforts that vary less than 20% was recorded^16^. The pulmonary function testing results were expressed as a percent of the predicted values for the Brazilian population^16^^-^ 19.

### Assessment of peripheral muscle strength

Muscle strength was assessed using an isometric dynamometer (model DIN-TRO, EMG System do Brasil LTDA, Brazil) and an endurance test under surface electromyography (EMG model 810C, EMG System do Brasil LTDA, Brazil). The participants were instructed to cross their arms over their chests, and the seat of the dynamometer was adjusted to allow for 90-degree hip flexion. The knees were at 90 degrees, and the dynamometer was positioned orthogonally to the longitudinal axis of the tibia, fixed to the ankle joint. The surface electromyography (EMG) electrodes were placed on the quadriceps (vastus medialis muscle) according to published recommendations^20^. Each participant performed three maximal voluntary isometric contractions (MVIC) with two-minute rest intervals, and the highest value was selected for analysis. The endurance test consisted of a 60-second sustained contraction at 30% of the MIVC measured in the strength test. A median frequency and root mean square slopes (MDF and RMS, respectively) corresponding to the EMG signal during the isometric contraction over time were used to analyze quadriceps fatigability^20^.

### Assessment of functional capacity

The degree of physical disability was assessed by means of the Health Assessment Questionnaire Disability Index (HAQ-DI) (short version). The HAQ-DI assesses an individual's degree of disability through 20 items distributed across eight domains of activities of daily living (dressing and grooming, arising, eating, walking, hygiene, reach, grip, and outside activity) that are scored from zero (without any difficulty) to three (unable to do). The disability index (DI) is calculated by adding the eight scores of the eight sections and then dividing the result by eight. The HAQ-DI has been validated for the Brazilian population^21^.

The 6MWT was performed in a 30-meter hallway. Heart rate, SpO2, and degree of dyspnoea on the modified Borg scale were measured before, at the third minute, and at the end of the test. The tests were repeated twice, and the highest value was recorded^12^. Each patient's predicted value was calculated by means of the equations formulated by Gibbons et al.^22^, as recommended by the American Thoracic Society^12^.

### Data analysis

To assess the homogeneity of the sample, the Shapiro-Wilk test was used; if a meaningful number of variables did not have a normal distribution, nonparametric tests were used. The results were expressed as the median and interquartile range values or as frequencies (percentages). The Mann-Whitney test was used to compare the SSc and the control groups. Spearman's rank correlations were calculated to investigate the associations between the peripheral and respiratory muscles measurements, the 6-min walk distance (6MWD), and quality of life parameters. Correlation coefficients <0.30 (or -0.30) represent little or no correlation; those in the range 0.30-0.49 (or -0.30--0.49) represent a weak correlation; those in the range 0.50-0.69 (or -0.50--0.69) represent a moderate correlation; those in the range 0.70-0.89 (or -0.70--0.89) represent a strong correlation; and those ≥0.90 (or -0.75) represent a strong correlation^23^. Data analysis was performed using SAS 6.11 software (SAS Institute, Inc., Cary, NC, USA). The statistical significance level was set at p<0.05.

## Results

Of the 31 SSc patients initially recruited, 11 were excluded for the following reasons: refusal to participate in the study (n=6), oxygen desaturation at rest (n=3), and difficulty in walking (n=2). Thus, the final sample included 19 women and one man in both the SSc group [53 (35.3-62.8) years old] and in the control group [46 (33.5-51.0) years old]. The median disease length was 5 (3-13.3) years. Ten patients exhibited the limited form of the disease and 10 exhibited the diffuse form. Arthralgia and complaints compatible with gastrointestinal and heart disease were reported by 10, nine, and eight individuals with SSc, respectively. Six patients reported using glucocorticoids in the past, five reported use of methotrexate, four of chloroquine, one of azathioprine, and one of cyclophosphamide. Three patients were ex-smokers.

Table 1 shows the comparisons of demographic data, body composition, pulmonary function, peripheral muscle performance, and functional capacity between the SSc and control groups. The SSc group exhibited lower FFM and fat percentage values; however, no significant differences were observed. Regarding peripheral muscle performance, the SSc group exhibited lower MDF and quadriceps strength values (p=0.034 and p=0.0001, respectively). The pulmonary function values and the 6MWD were significantly lower in the SSc group. Table 2 shows the results of the quality of life and physical capacity questionnaires between the SSc and control groups. The SSc group exhibited lower SF-36 physical component summary and SF-36 mental component summary scores (p=0.006 and p=0.03, respectively) and higher HAQ-DI value (p=0.007).

**Table 1.** Comparison of demographic data, body composition, lung function, peripheral muscle performance, and functional capacity of healthy volunteers and SSc patients.

	Control group (n=20)	SSc group (n=20)	p*-*value
**Demographic data**			
Age (years)	46 (33.5–51.0)	53 (35.3–62.8)	0.39
Sex (female)	19 (95%)	19 (95%)	1.00
Height (cm)	160.1±5.87	157.5±6.55	0.21
**Body composition**			
Weight (kg)	65.2±7.76	61.1±9.23	0.23
Body mass index (kg/m^2^)	25.2 (23.3–29.8)	23.3 (19.3–27.7)	0.14
FFM (kg)	40.3 (24.6–46.8)	36.3 (29.7–40.7)	0.81
Fat percentage (%)	34.8 (27.8–39.3)	32.6 (21.5–37.8)	0.29
**Lung function**			
FVC (% predicted)	101.5 (90.8–106.8)	73.5 (58.8–103.8)	0.011
DLco (% predicted)	102 (94.5–106.5)	66.5 (47.5–87.8)	0.0001
MIP (% predicted)	95 (83.5–107.5)	53 (44.3–65.8)	0.0001
MEP (% predicted)	79 (72.8–87.5)	38 (30.3–52.5)	0.0001
**Peripheral muscle performance**			
RMS slope	0.32 (0.12–0.66)	0.64 (0.18–1.21)	0.15
MDF slope	-0.07 (-0.19–-0.01)	-0.17 (-0.41–-0.09)	0.034
Quadriceps strength (kg)	36 (32–41.4)	23.1 (18.1–31.2)	0.0001
6MWD (% predicted)	109.5 (96.3–137.9)	83.5 (73.2–99)	0.0001

Values are medians (interquartile range) or number (%). FFM = fat-free mass; FVC = forced vital capacity; DLco = diffusing capacity for carbon monoxide; MIP = maximal inspiratory pressure; MEP = maximal expiratory pressure; RMS = angle of the linear regression line obtained with the values of root mean square electromyography signal over time during fatigability test of vastus medialis muscle; MDF = angle of the linear regression line obtained with the values of median frequency electromyography signal over time during the fatigability test of vastus medialis muscle; 6MWD = 6-minute walk distance.

**Table 2.** Health-related quality of life and functional status (disability) measures in SSc patients.

	Control group (n=20)	SSc group (n=20)	p*-*value
**SF-36**			
Physical functioning	95 (90−96.2)	50 (21.3−73.8)	0.0001
Physical role	100 (75−100)	25 (0−75)	0.007
Bodily pain	62 (51.7−79)	51 (40.3−74)	0.23
General health perception	62 (52−68.2)	57 (33−62)	0.23
Vitality	65 (55−76.2)	45 (27.5−63.8)	0.10
Social functioning	100 (78.1−100)	56.3 (37.5−87.5)	0.01
Emotional role	100 (91.7−100)	16.7 (0−91.7)	0.002
Mental health	68 (52−85)	62 (40−71)	0.17
SF-36 PCS	47 (45.7−52.8)	40.6 (28.6−46.1)	0.006
SF-36 MCS	50 (44−58.1)	40.6 (29.9−48.5)	0.03
**HAQ-DI**	0.03 (0−0.16)	0.75 (0.04−1.27)	0.007

Values are medians (interquartile range) or number (%). SF-36 = Medical Outcomes Study 36-Item Short-Form Health Survey; PCS = Physical component summary score; MCS = physical component summary score; HAQ-DI = Health Assessment Questionnaire Disability Index.

**Figure 1.**
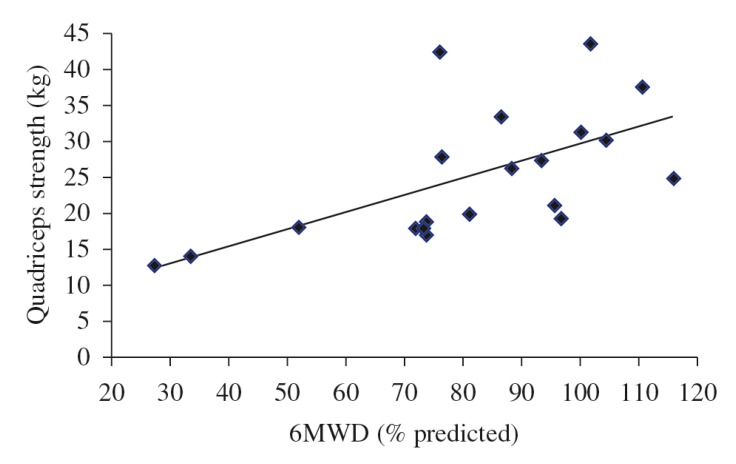
Relationship between quadriceps strength and the 6-min walk distance (6MWD) (Rho=0.719; p=0.0004).

**Figure 2.**
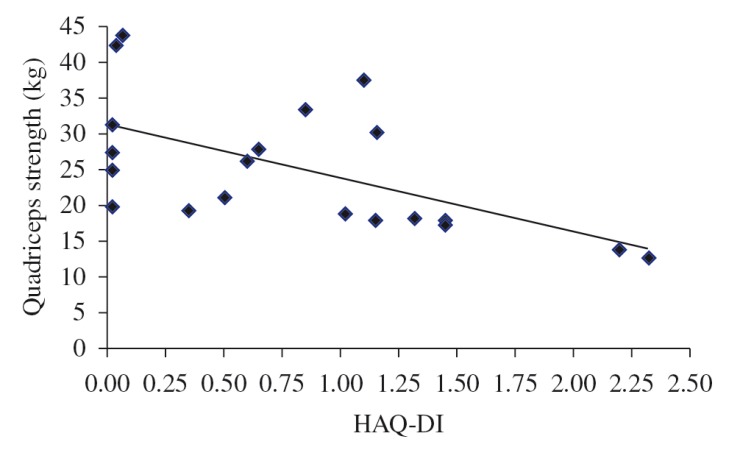
Relationship between quadriceps strength and the Health Assessment Questionnaire Disability Index (HAQ-DI) (Rho=-0.622; p=0.003).

The results of the univariate correlation analysis of the peripheral and respiratory muscle assessment, functional capacity, and quality of life in SSc patients are shown in Table 3. Quadriceps strength exhibited a strong correlation with the 6MWD. Quadriceps strength exhibited a moderate correlation with the HAQ-DI. The correlations of quadriceps strength with the 6MWD and the HAQ-DI are shown in Figures 1 and 2. The MDF exhibited a moderate correlation with MIP (*Rho*=0.684; p=0.0009) and a weak correlation with MEP (*Rho*=0.472; p=0.035). No significant correlations between these variables were found in healthy subjects.

**Table 3.** Spearman's correlation coefficients between peripheral and respiratory muscles performance, functional capacity, and quality of life in SSc patients.

	RMS	MDF	Quadriceps strength	MIP	MEP
6MWD	0.054	0.116	0.719^†^	0.072	0.298
SF-36 PCS	0.143	0.111	0.045	0.163	0.135
SF-36 MCS	-0.044	-0.056	-0.096	-0.010	0.046
HAQ-DI	-0.084	0.017	-0.622^*^	0.282	0.235

RMS = angle of the linear regression line obtained with the values of root mean square electromyography signal over time during fatigability test of vastus medialis muscle; MDF = angle of the linear regression line obtained with the values of median frequency electromyography signal over time during the fatigability test of vastus medialis muscle; MIP = maximal inspiratory pressure; MEP = maximal expiratory pressure; 6MWD = 6-minute walk distance; SF-36 = Medical Outcomes Study 36-Item Short-Form Health Survey; PCS = physical component summary score; MCS = mental component summary score; HAQ-DI = Health Assessment Questionnaire Disability Index (HAQ-DI).

* p<0.01;

† p<0.001.

## Discussion

The main findings in the present study are as follows: (1) patients with SSc exhibited a reduction in the strength of the quadriceps and an increase in its fatigability, as well as reduced functional capacity; (2) peripheral muscle strength exhibited a significant correlation with the 6MWD and the HAQ-DI; and (3) fatigability exhibited a relationship with MIP and MEP. So far, no other study has focused on the association of objective measures of lower limb muscle performance with the functional capacity and quality of life of individuals with SSc.

Compared to the control group, the SSc patients exhibited a reduction in the strength of the quadriceps and an increase in its fatigability. We were not able to locate any other study in the literature that assessed individuals with SSc by using isometric dynamometry and endurance testing of the quadriceps muscle. However, the characteristics of muscle affection in SSc are well established. According to Deuschle et al.^5^, muscle biopsies of SSc patients show an increase in the connective tissue in the epimysium and perimysium, along with microangiopathy^5^. Interestingly, a higher frequency of heart disease has been reported in association with SSc-related myopathy^24^, which suggests that muscle affection is systemic and generalized in SSc patients. In accordance with other authors^5^^,^ 6 ^,^ 10, we also found a reduced 6MWD in patients with SSc. The contribution of the muscular system impairment to the low aerobic capacity and poor exercise performance may be important, even in individuals with subclinical muscle affection^10^.

Similar to other studies^14^^,^ 25, we also observed lower FFM and fat percentage values in patients with SSc compared to controls, but no significant differences. Reduced FFM in individuals with SSc may be attributed to several factors, including reduced physical activity, malnutrition, intestinal malabsorption, and use of glucocorticoids^10^. In addition to contributing to the pathogenesis of malnutrition, inflammation bears a close relationship with muscle wasting^14^^,^ 25, which at least partially explains the moderate correlation between FFM and quadriceps strength found in the present study. Therefore, we believe that individuals with SSc should be encouraged to improve their nutritional status and physical performance. Future prospective studies may contribute to the assessment of the effects of nutritional support and physical reconditioning on patients with SSc.

Exercise testing remains a robust and versatile tool that provides valuable diagnosis and prognostic information^11^^,^ 22. In patients with SS who frequently experience musculoskeletal pain, the 6MWT may be more feasible and acceptable than tests that involve exercise equipment, such as the cycloergometer or treadmill. However, whether muscle affection in SSc patients exerts an impact on the 6MWT is still an open question. In the present study, we found a strong correlation between quadriceps strength and the 6MWD (*Rho*=0.719; p=0.0004). In addition to myositis and myopathy, it is also believed that changes in the small blood vessels of skeletal muscles may exert an influence on the oxygen supply to cells, thus contributing to poorer exercise performance^8^^,^ 24.

The HAQ-DI is a musculoskeletal-targeted questionnaire that assesses the impact of SSc on physical functioning and disability^26^. The DI values we found are close to those reported by Singh et al.^26^ and de Achaval et al.^27^, but diverge from those reported by Brower and Poole^28^ and Rannou et al.^29^, which possibly reflects differences in the severity of SSc among the patients tested. We also observed a moderate correlation between the HAD-QI values and quadriceps strength as reported by Steen and Medsger Jr.^30^, who applied clinical criteria to define the presence of proximal muscle weakness. According to Rannou et al.^29^, the HAQ-DI should be preferred over the SSc HAQ (sHAQ) for assessing physical functioning, and the physical and mental component summary measures of the SF-36 are questionable for SSc patients.

In addition to reduced strength, we also found greater fatigability in the quadriceps muscle in the individuals with SSc. According to Hunzelmann and Brinckmann^31^, hypoxia is a decisive factor for modulating the inflammatory process in SSc, activating fibroblasts and changing their phenotype. The fact that type II fibers exhibit easy fatigability in the presence of hypoxia may contribute to the reduced muscle endurance in individuals with SSc. We also observed a relationship between quadriceps fatigability and measures of respiratory muscle strength, which suggests that muscle weakness is generalized in individuals with SSc.

The strongest point of the present study is that it demonstrates that there are functional differences between SS patients and healthy subjects and that, although many factors have the potential to interfere with the functional capacity of SS patients, muscle strength has a strong association with performance in 6MWD. In addition, it is the first study to investigate the correlations between dynamometry parameters and nutritional status, pulmonary function, and functional capacity in these individuals. Patients' results were compared to those from a control group matched according to gender, age, height, weight, and BMI. We consider the small sample size to be the main limitation of our study. Moreover, we did not measure serum markers such as creatine kinase/aldolase and did not conduct magnetic resonance imaging analysis or histological analysis of biopsy specimens, which are useful for assessing muscle abnormalities. However, we believe that the results of the present study justify the need for further research on the peripheral muscle strength of patients with SSc in order to improve the understanding of the pathophysiological mechanisms of the disease.

To conclude, the present study showed that patients with SSc exhibited reduced respiratory muscles and quadriceps strength and an increase in its fatigability. In addition, quadriceps strength/endurance exhibited a relationship with the functional capacity and quality of life of these individuals. Although the nature of our results requires a more thorough investigation, our data suggest that quadriceps strength/endurance should be assessed in clinical practice.

## References

[1] Nikpour M, Stevens WM, Herrick AL, Proudman SM (2010). Epidemiology of systemic sclerosis. Best Pract Res Clin Rheumatol.

[2] Barnabe C, Joseph L, Belisle P, Labrecque J, Edworthy S, Barr SG (2012). Prevalence of systemic lupus erythematosus and systemic
sclerosis in the First Nations population of Alberta, Canada. Arthritis Care Res (Hoboken).

[3] Mouthon L (2013). [Hand involvement in systemic sclerosis]. Presse Med.

[4] Krause L, Becker MO, Brueckner CS, Bellinghausen CJ, Becker C, Schneider U (2010). Nutritional status as marker for disease activity and
severity predicting mortality in patients with systemic sclerosis. Ann Rheum Dis.

[5] Deuschle K, Weinert K, Becker MO, Backhaus M, Huscher D, Riemekasten G (2011). Six-minute walk distance as a marker for disability and
complaints in patients with systemic sclerosis. Clin Exp Rheumatol.

[6] Holland AE, Goh NS (2012). The six-minute walk test in scleroderma: what should we
measure and how should we measure it?. Respirology.

[7] Medsger TA Jr, Rodnan GP, Moossy J, Vester JW (1968). Skeletal muscle involvement in progressive systemic
sclerosis (scleroderma). Arthritis Rheum.

[8] Clements PJ, Furst DE, Campion DS, Bohan A, Harris R, Levy J (1978). Muscle disease in progressive systemic sclerosis:
diagnostic and therapeutic considerations. Arthritis Rheum.

[9] Ranque B, Bérezné A, Le-Guern V, Pagnoux C, Allanore Y, Launay D (2010). Myopathies related to systemic sclerosis: a case-control
study of associated clinical and immunological features. Scand J Rheumatol.

[10] Azevedo VF, Müller CS, Rinaldi L, Bredt MC, Giovanni K, Pereira MAC (2009). Avaliação nutricional e da capacidade funcional em
doentes com esclerose sistémica progressiva. Acta Reumatol Port.

[11] ATS Committee on Proficiency Standards for Clinical Pulmonary Function
Laboratories (2002). ATS statement: guidelines for the six-minute walk
test. Am J Respir Crit Care Med.

[12] Schoindre Y, Meune C, Dinh-Xuan AT, Avouac J, Kahan A, Allanore Y (2009). Lack of specificity of the 6-minute walk test as an
outcome measure for patients with systemic sclerosis. J Rheumatol.

[13] van den Hoogen F, Khanna D, Fransen J, Johnson SR, Baron M, Tyndall A (2013). 2013 classification criteria for systemic sclerosis: an
American College of Rheumatology/European League against Rheumatism collaborative
initiative. Arthritis Rheum.

[14] Ciconelli RM, Ferraz MB, Santos W, Meinão I, Quaresma MR (1999). Brazilian-Portuguese version of the
SF-36. A reliable and valid quality of life outcome measure. Rev Bras
Reumatol.

[15] Kyle UG, Bosaeus I, De Lorenzo AD, Deurenberg P, Elia M, Gómez JM, et al, Composition of the ESPEN Working Group (2004). Bioelectrical impedance analysis-part I: review of
principles and methods. Clin Nutr.

[16] Miller MR, Hankinson J, Brusasco V, Burgos F, Casaburi R, Coates A (2005). Standardization of spirometry. Eur Respir J.

[17] Pereira CAC, Sato T, Rodrigues SC (2007). New reference values for forced spirometry in white
adults in Brazil. J Bras Pneumol.

[18] Neder JA, Andreoni S, Lerario MC, Nery LE (1999). Reference values for lung function tests. II. Maximal respiratory pressures and voluntary ventilation. Braz J Med Biol
Res.

[19] Neder JA, Andreoni S, Peres C, Nery LE (1999). Reference values for lung function tests. III. Carbon
monoxide diffusing capacity (transfer factor). Braz J Med Biol Res.

[20] Hermens HJ, Freriks B, Disselhorst-Klug C, Rau G (2000). Development of recommendations for SEMG sensors and
sensor placement procedures. J Electromyogr Kinesiol.

[21] Shinjo SK, Gonçalves R, Kowalski S, Gonçalves CR (2007). Brazilian-Portuguese version of the Health Assessment
Questionnaire for Spondyloarthropathies (HAQ-S) in patients with ankylosing
spondylitis: a translation, cross-cultural adaptation, and
validation. Clin Rheumatol.

[22] Gibbons WJ, Fruchter N, Sloan S, Levy RD (2001). Reference values for a multiple repetition 6-minute walk
test in healthy adults older than 20 years. J Cardiopulm Rehabil.

[23] Baptista CR, Costa AA, Pizzato TM, Souza FB, Mattiello-Sverzut AC (2014). Postural alignment in children with Duchenne muscular
dystrophy and its relationship with balance. Braz J Phys Ther.

[24] Mimura Y, Ihn H, Jinnin M, Asano Y, Yamane K, Tamaki K (2005). Clinical and laboratory features of scleroderma patients
developing skeletal myopathy. Clin Rheumatol.

[25] Souza RB, Borges CT, Takayama L, Aldrighi JM, Pereira RM (2006). Systemic sclerosis and bone loss: the role of the
disease and body composition. Scand J Rheumatol.

[26] Singh MK, Clements PJ, Furst DE, Maranian P, Khanna D (2012). Work productivity in scleroderma: analysis from the
University of California, Los Angeles scleroderma quality of life
study. Arthritis Care Res (Hoboken).

[27] de Achaval S, Kallen MA, Mayes MD, Lopez-Olivo MA, Suarez-Almazor ME (2013). Use of the Patient-generated Index in systemic sclerosis
to assess patient-centered outcomes. J Rheumatol.

[28] Brower LM, Poole JL (2004). Reliability and validity of the Duruoz Hand Index in
persons with systemic sclerosis (scleroderma). Arthritis Rheum.

[29] Rannou F, Poiraudeau S, Berezné A, Baubet T, Le-Guern V, Cabane J (2007). Assessing disability and quality of life in systemic
sclerosis: construct validities of the Cochin Hand Function Scale, Health
Assessment Questionnaire (HAQ), Systemic Sclerosis HAQ, and Medical Outcomes Study
36-Item Short Form Health Survey. Arthritis Rheum.

[30] Steen VD, Medsger TA Jr (1997). The value of the Health Assessment Questionnaire and
special patient-generated scales to demonstrate change in systemic sclerosis
patients over time. Arthritis Rheum.

[31] Hunzelmann N, Brinckmann J (2010). What are the new milestones in the pathogenesis of
systemic sclerosis?. Ann Rheum Dis.

